# Deep Epicardial Laceration after Cardiopulmonary Resuscitation: A Case Report

**DOI:** 10.5041/RMMJ.10455

**Published:** 2021-10-25

**Authors:** Noa Fried Regev, Tzachi Slutsky, Oren Lev-Ran, Yaron Ishai, Dan Schwarzfuchs

**Affiliations:** 1 Department of Emergency Medicine, Soroka University Medical Center, Beer-Sheva, Israel; 2 Department of Cardiothoracic Surgery, Soroka University Medical Center, Beer-Sheva, Israel

**Keywords:** Cardiopulmonary resuscitation, epicardial laceration

## Abstract

Effective chest compressions have been proven to be a key element in a successful cardiopulmonary resuscitation (CPR). However, unintended injuries have been described in the medical literature for decades, including major intrathoracic injuries. We present a case of an 80-year-old man after a successful CPR who was later diagnosed with deep epicardial laceration as a result of effective chest compressions.

## CASE PRESENTATION

**Figure 1:**
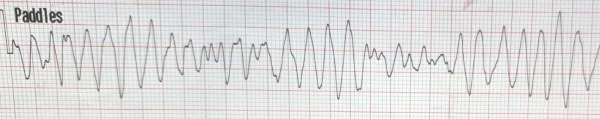
Ventricular Fibrillation as Seen on the Monitor upon the Patient’s Arrival to the Emergency Room.

**Figure 2:**
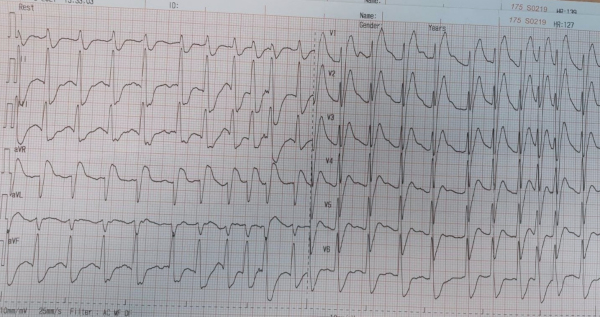
Electrocardiogram of Atrial Fibrillation after Two Shocks during Resuscitation.

An 80-year-old man with Marfan syndrome and no previous cardiac history collapsed on hospital grounds after complaining of bilateral shoulder pain. Within a few minutes, two doctors at the scene started performing chest compressions. The patient was immediately transferred to the emergency room where he was intubated and defibrillated twice due to ventricular fibrillation (Figure 1), with return of spontaneous circulation (ROSC) in between the two shocks (Figure 2). During resuscitation, he was treated with adrenaline and amiodarone.

After a second ROSC, cardiac rhythm converted to atrial fibrillation with ST segment elevation in the leads corresponding to the anterior wall. As part of the treatment, he received magnesium 2 g, heparin bolus 5000 IU, and 300 mg aspirin by nasogastric tube.

**Figure 3:**
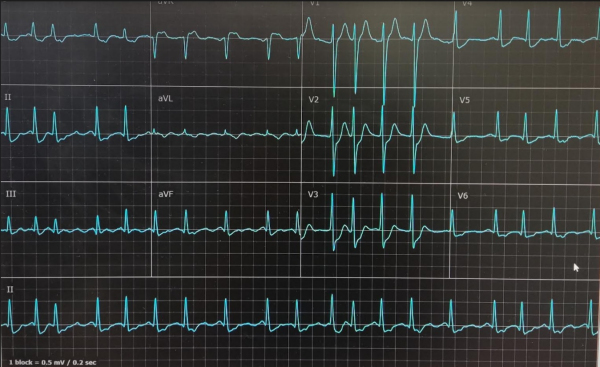
Electrocardiogram of Atrial Fibrillation with Resolution of ST Segment Elevation and Diffuse ST Segment Depression after Cardioversion in the Intensive Coronary Care Unit.

**Figure 4:**
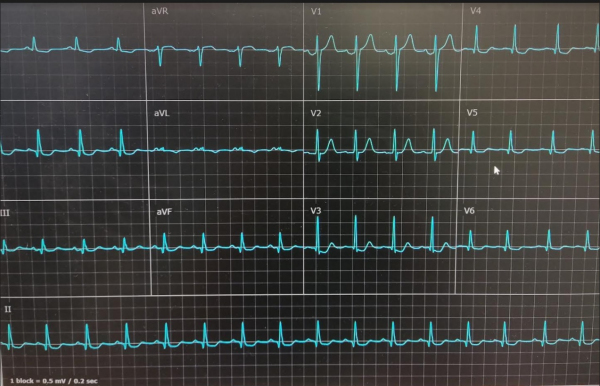
Electrocardiogram of Normal Sinus Rhythm with Diffuse ST Segment Depression after Cardioversion in the Intensive Coronary Care Unit.

The patient was admitted to the intensive coronary care unit. On admission, the electrocardiogram showed rapid atrial fibrillation with ST segment ele-vation resolution (Figure 3). Due to hemodynamic compromise, electrical cardioversion was performed, converting to sinus rhythm with diffuse ST segment depression and an ST elevation in lead aVR (Figure 4). The patient was taken urgently to the cardiac catheterization laboratory due to ST changes and hemodynamic instability. The angiography demon-strated significant two-vessel disease, and a drug-eluting stent was implanted successfully to the proximal to mid-left anterior descending artery (LAD).

During the early post-procedural period the patient became hemodynamically unstable; bedside echocardiography demonstrated accumulation of a new pericardial effusion. The differential diagnosis included coronary artery perforation versus cardiac laceration. After a brief discussion with the heart team, the patient was transferred for a second time to the catheterization laboratory in order to implant a cover stent in case there was indeed a coronary perforation and pericardiocentesis was needed for the accumulated pericardial effusion.

During angiography, instant stent thrombosis was detected. The LAD was re-opened, and no coronary perforation was found. Post-intervention transthoracic echocardiography showed no significant changes. There was mild to moderate pericardial effusion and no signs of cardiac tamponade.

Because of the complexity of the coronary intervention, another injection of iodine-containing contrast media to the LAD was performed, which revealed total occlusion due to stent thrombosis. At this point it was decided to perform emergency coronary artery bypass grafting (CABG) surgery due to failed percutaneous intervention.

**Figure 5:**
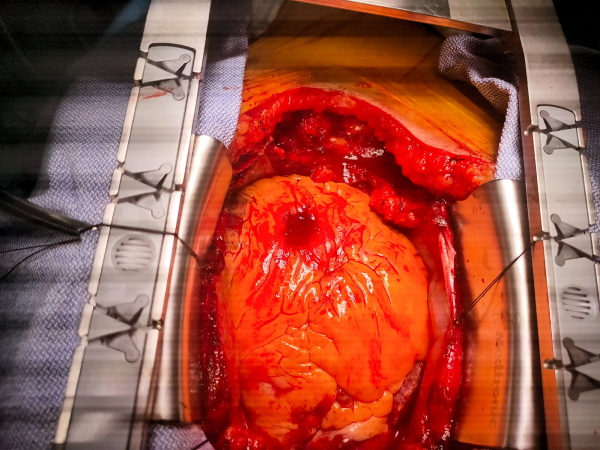
Deep Laceration to the Epicardium above the Right Ventricle during Open Heart Surgery.

Midsternotomy incision was performed, and sternal fractures were discovered. There was a significant amount of blood upon entry into the chest cavity, with clotted blood in the retrosternal and mediastinal fat. The source of bleeding was identified as a deep laceration to the epicardium above the right ventricle (Figure 5), caused by an injured blood vessel (and not a complete perforation of the myocardium as was initially assumed). This injury was assumed to be spatially related to fractures of the sternum.

**Figure 6:**
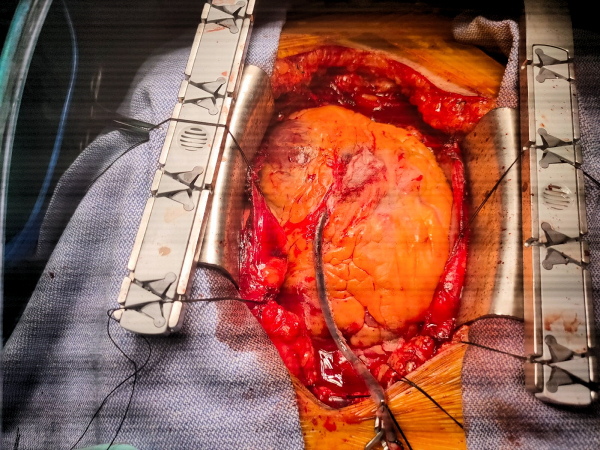
Laceration after Repair with an Epicardial Patch during Open Heart Surgery.

The laceration was repaired with a pericardial patch (Figure 6). An “off pump” CABG (without cardiopulmonary bypass) procedure was performed with the anastomoses of the saphenous vein to mid LAD artery and to the aorta. The sternal fractures were fixated.

The patient was then transferred to the Cardio-Thoracic Intensive Care Unit.

## DISCUSSION

Effective chest compressions have been proven to be a key element for successful cardiopulmonary resuscitation (CPR).^1^ However, the repetitive compressions to the chest wall may also cause complications. Unintended injuries have been described in the medical literature for decades.^2^ Most frequently reported are skeletal injuries, specifically rib and sternum fractures. Major life-threatening intrathoracic injuries, such as to the heart and great vessels, are much less common.^3^

Unfortunately, various studies have relied almost entirely on postmortem examinations, and data on CPR injuries in survivors of cardiac arrest remain limited.^2^

We present a rare case of a patient undergoing successful CPR, in a fascinating chain of events that led to diagnosis during open-heart surgery of a deep laceration to the epicardium. This case highlights the risk of life-threatening intrathoracic injuries such as epicardial laceration caused by effective and forceful chest compressions during CPR, and the importance of closely monitoring post-resuscitation patients using different imaging modalities, including bedside echocardiography.

## Abbreviations

CABG: coronary artery bypass grafting
CPR: cardiopulmonary resuscitation
LAD: left anterior descending artery
ROSC: return of spontaneous circulation.
